# Supplementary material for: Plasmonic nano-aperture label-free imaging (PANORAMA)
Source: Nat Commun. 2020 Nov 16;11:5805. doi: 10.1038/s41467-020-19678-w (PMC7670455; doi:10.1038/s41467-020-19678-w)
Supplement: Supplementary file 1 — Supplementary Information [file 41467_2020_19678_MOESM1_ESM.pdf]

SUPPLEMENTARY INFORMATION

Plasmonic nano-aperture label-free imaging

Nareg Ohannesian[1], Ibrahim Misbah[1], Steven H. Lin[5], and Wei-Chuan Shih[1,2,3,4,*]

1 Department of Electrical and Computer Engineering, University of Houston, 4800 Calhoun Road, Houston, Texas 77204, United States of America
2 department of Biomedical Engineering, University of Houston, 4800 Calhoun Road, Houston, Texas 77204, United States of America
3 Department of Chemistry, University of Houston, 4800 Calhoun Road, Houston, Texas 77204, United States of America
4 Program of Materials Science and Engineering, University of Houston, 4800 Calhoun Road, Houston, Texas 77204, United States of America
5 Department of Radiation Oncology, The University of Texas MD Anderson Cancer Center, Houston, Texas, United States of America
[*]E-mail: wshih@uh.edu

Supplementary Note 1: AGNIS substrates properties

AGNIS consists of a two-dimensional polycrystalline array of gold nanodisks with an average diameter of 360 nm with a pitch size (center-to-center distance) of 460 nm (Supplementary Figure 1a). This gold nanodisk array was undercut in a buffer HF solution to partially remove glass substrate beneath the disks. Due to the 100 nm edge to edge distance of nanodisk, it is difficult to image the undercut portion via SEM. Instead, we use a similar substrate to AGNIS but with an edge to edge distance of 500 nm where the undercut portion is easily visible (Supplementary Figure 1b). The LSPR extinction peak of AGNIS is at 620 nm in air and 690 nm in water (Supplementary Figure 1c). Fig. S1d shows the LSPR peak blue shifted from 830 nm to 690 nm after the substrate undercut (Supplementary Figure 1d).

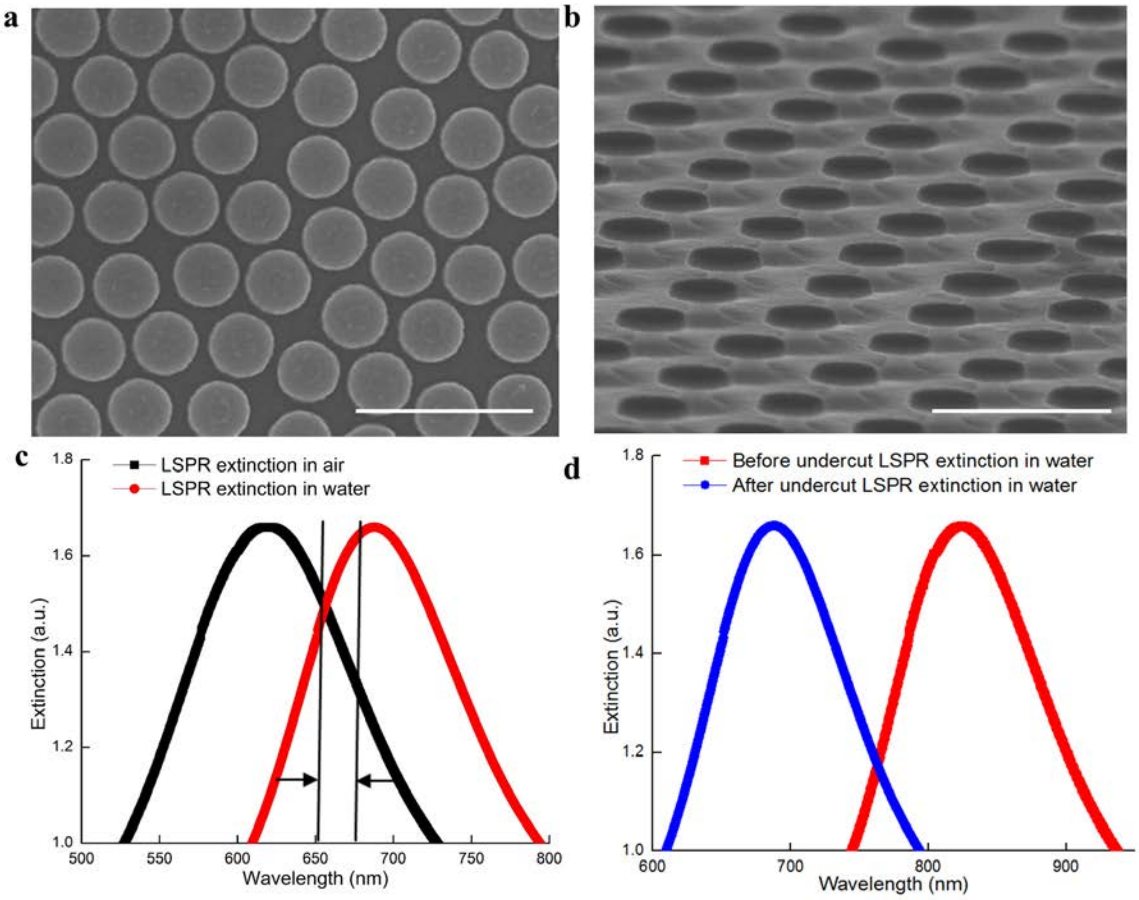

Supplementary Figure 1: **a**, SEM image of AGNIS used to perform PANORAMA; **b**, SEM image of AGNIS with an edge to edge disk distance of 500 nm to reveal the undercut glass substrate. **c**, LSPR extinction curve of bare AGNIS in different media alongside the imaging wavelength range labeled with arrows. **d**, LSPR peak in water blue shifts from 830 nm to 690 nm after substrate undercut. Scale bar: 1 µm.

Supplementary Note 2: System setup

White light from a tungsten-halogen lamp passes through a condenser (IX-LWUCD, Olympus) and illuminates the sample on an inverted microscope (IX71, Olympus). The transmitted light passes through an infinity corrected 60X water immersion lens with a 1.2 numerical aperture (UPLSAPO60XW, Olympus). The light exiting the side-port is relayed to an electron multiplied charge coupled device (EMCCD; ProEM 1024, Princeton Instruments) via a 4f system (Supplementary Figure 2, L1, and L2), where a bandpass filter with 650-670 nm passband (FB660-10, Thorlabs) at its Fourier plane.

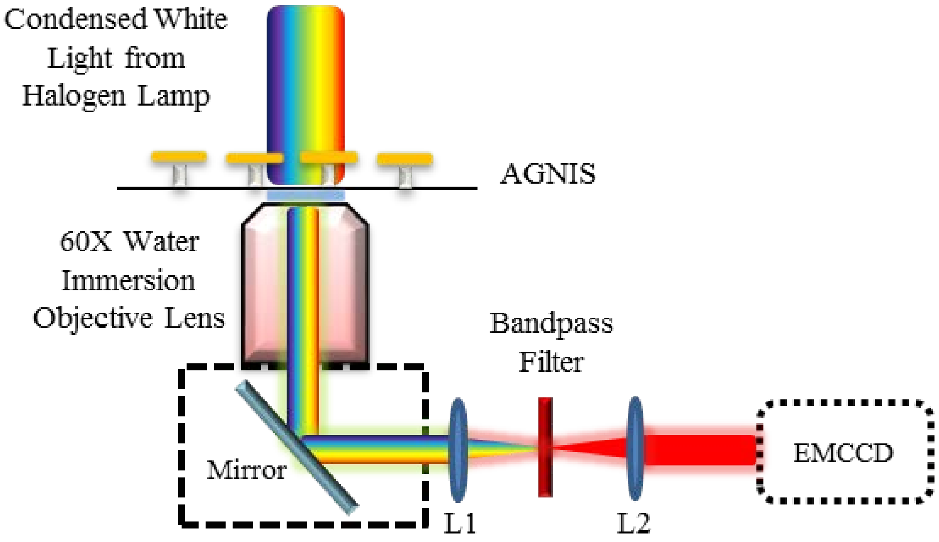

Supplementary Figure 2: PANORAMA system setup.

Supplementary Note 3: Correlation of PANORAMA with LSPR extinction peak shift

The amount of light transmitted through the nano-aperture is governed by the LSPR extinction peak shift. Thus, a larger LSPR red shift leads to a larger intensity ratio (IR, defined in the paper). To examine this statement that PANORAMA signal is indeed due to the LSPR red shift, we have performed finite difference time domain (FDTD) simulations with different sized PS nanoparticles on the AGNIS. The results are plotted against the IR values from experimental results. As shown in Supplementary Figure 3, the experimentally obtained IR values correlate well with the LSPR red shift.

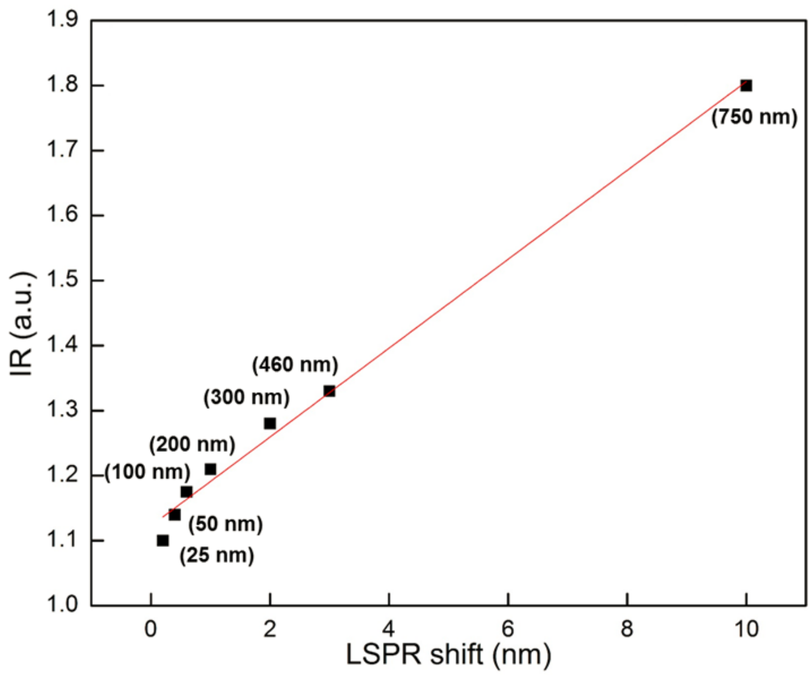

Supplementary Figure 3: Intensity ratio (IR) obtained from experimental data vs. LSPR shift from FDTD simulations with PS nanoparticle diameter indicated in parenthesis.
